# N,N′-Dinitrosopiperazine-Mediated AGR2 Is Involved in Metastasis of Nasopharyngeal Carcinoma

**DOI:** 10.1371/journal.pone.0092081

**Published:** 2014-04-09

**Authors:** Yuejin Li, Jinping Lu, Zhengke Peng, Gongjun Tan, Na Liu, Damao Huang, Zhenlin Zhang, Chaojun Duan, Xiaowei Tang, Faqing Tang

**Affiliations:** 1 Clinical Laboratory and Medical Research Center, Zhuhai Hospital, Jinan University, Zhuhai People’s Hospital, Zhuhai, Guangdong, People’s Republic of China; 2 Medical Research Center and Clinical Laboratory, Xiangya Hospital, Central South University, Changsha, Hunan, People’s Republic of China; 3 Metallurgical Science and Engineering, Central South University, Changsha, People’s Republic of China; South China Sun Yat-sen University Cancer Center, China

## Abstract

Nasopharyngeal carcinoma (NPC) has a high metastatic character in the clinic, but its mechanism is not clear. As a carcinogen with organ specificity for the nasopharyngeal epithelium, N,N′-Dinitrosopiperazine (DNP) is involved in NPC metastasis. Herein, our data revealed that anterior gradient 2 (AGR2) was overexpressed in human NPC tissues, particularly in cervical lymph node metastatic NPC (LMNPC). High AGR2 expression was associated with NPC metastasis. Importantly, DNP induced AGR2 expression, and increased cell motility and invasion in the NPC cell line 6–10B. However, DNP-mediated cell motility and invasion was dramatically decreased when transfected with siRNA-AGR2. Further, AGR2 directly regulated cathepsin (CTS) B and D by binding them *in vitro*. These results indicate that DNP induces AGR2 expression, regulates CTSB and CTSD, increases cell motility and invasion, and promotes NPC tumor metastasis. Therefore, DNP-mediated AGR2 expression may be an important factor in prolific NPC metastasis.

## Introduction

Nasopharyngeal carcinoma (NPC) is a common malignant cancer in southern China [1]. Epidemiological investigations have revealed that the incidence of NPC has remained high in endemic regions, particularly in Southeast Asia, with an incidence of 30–80 per 100,000 people per year in southern China [2]. In spite of significant advances in early diagnosis, surgical intervention, as well as local and systemic adjuvant therapies, the majority of NPC deaths are attributable to tumor invasion and distant metastases that are resistant to available therapies [3]–[5] .

In endemic NPC, >95% are classified as undifferentiated World Health Organization (WHO) type III and is universally associated with the Epstein–Barr virus (EBV) [6], a strong etiological factor interacting with genetic predisposition [7], and dietary intake of preserved foods [8]. Moreover, in studies on Chinese populations in high-incidence regions, the relative risk of NPC is related to the region’s eating habits, particularly with dietary intake of salt-preserved fish [8]–[15] . The process of salt preservation is inefficient and the food can become partially putrefied. Consequently, these foods accumulate significant levels of nitrosamines [16], [17], which are known carcinogens [16], [18], [19]. N,N′-Dinitrosopiperazine (DNP) is one predominant volatile nitrosamine in salted fish [13], [20]. The carcinogenic potential of DNP in salt-preserved fish is supported by experiments in rats, which develop malignant nasal and NPC [21]–[23]. Furthermore, DNP can induce malignant transformations of human embryonic nasopharyngeal epithelial cells [24].

DNP-induced NPC, is organ specific to the nasopharyngeal epithelium, and is involved in nasopharyngeal tumorigenesis, motility and invasion [25], [26], [57]. Our recent work indicates that DNP induces NPC metastasis, following high expression of anterior gradient 2 (AGR2), cathepsin B (CTSB), and cathepsin D (CTSD) [27]. AGR2 is upregulated in multiple cancers, including breast [28], lung adenocarcinoma [29], [30], ovarian [31], and prostate cancers [32]. Its high expression is associated with tumor metastasis and poor prognosis [33], while silencing AGR2 inhibits cell growth and cell cycle progression, and induces cell death [34]. The mechanism linking AGR2 to tumor invasion and metastasis is involved in several molecular alterations such as CTSB and CTSD [35]. CTSB and CTSD degrade extracellular matrix proteins, and recently they have been implicated in cancer invasion and metastasis. As a lysosomal cysteine endoproteinase, CTSB can degrade laminin, fibronectin, collagen, and other extracellular matrix components, and promote the formation of tumor blood vessels; therefore, it is believed to be one of the key enzymes in invasion and metastasis of malignant tumors. It is upregulated in glioma [36], laryngeal cancer [37], cervical cancer [38], [39], bladder cancer [40], gastrointestinal tract cancer [41]–[43], oral carcinoma [44], and breast cancer [45]–[47], and its expression level is correlated to metastatic potential. CTSD, a lysosomal aspartate proteolytic enzyme, also plays a role in invasion and metastasis of cancer. It is upregulated in some malignant tumor metastases [48], gastric cancer with lymphatic and/or blood vessel invasion [49], breast cancer [50], [51], colorectal cancer [52], [53], liver cancer [54] and pancreatic cancer [55]. Furthermore, Cheng et al [56] observed significant CTSD upregulation in lymph node metastasis versus primary NPC, which was significantly correlated with advanced clinical stage, recurrence, and lymph node and distant metastasis.

To explore the role and possible mechanism of DNP-induced invasion and metastasis, we first detected the expression of AGR2, CTSB, and CTSD in metastatic NPC tissues. We then investigated the mechanism of AGR2 in DNP-induced NPC invasion and metastasis. We found that DNP induces AGR2 expression. AGR2 then binds to CTSB and CTSD, and promotes NPC cell motility and invasion leading to NPC metastasis.

## Materials and Methods

### Ethics Statement

This project’s experimental designs and protocol were submitted to the ethical committee at Zhuhai Hospital of Jinan University and Xiangya Hospital of Central South University before performing the study. The ethical committee members reviewed the experimental designs and protocols to determine whether these studies would hurt the security and privacy of the patients enrolled, and gave ethical approval. Additionally, all patients enrolled agreed to participate in the project and gave signed consent.

### Cell Lines and Tissues

The human NPC cell line 5–8F and 6–10B (sublines derived from cell line SUNE-1) were purchased from the Cancer Research Center of Sun Yatsen University (Guangzhou, China). The 5–8F cell has a high metastatic ability. The 6–10B cell has a low metastatic ability [26]. The Cell lines were cultured as a monolayer in RPMI 1640 medium containing 10% fetal bovine serum (FBS), 2 mM l-glutamine, 100 μg/mL penicillin, and 100 IU/mL streptomycin (Invitrogen, Carlsbad, CA), and were maintained in an incubator with a humidified atmosphere of 95% air and 5% CO2 at 37°C. For DNP treatment, DNP crystals were dissolved in dimethyl sulfoxide (DMSO), and appropriate amounts of the DNP stock solution were added to the culture cells to achieve the indicated concentrations. The cells were then incubated for the indicated times. To investigate the dose-course of DNP treatment, 6–10B cells were treated with 0, 2, 6, 8, or 10 μM DNP for 24 h. For time-course assays, the cells were treated with 8 μM DNP for 0, 6, 18, 24, 36, or 48 h.

One hundred and thirty-four tissue specimens, including 60 cases of primary NPC, 48 normal nasopharyngeal epithelial tissues (NNET), and 26 cervical lymph node metastatic NPC (LMNPC), were obtained from the Xiangya Hospital of Central South University from January 2010 to September 2012. Each specimen was fixed with 40 g/L paraformaldehyde solution, followed by dehydration and paraffin embedment. Four-microgram serial sections were utilized for immunohistochemistry. All patients had only one primary tumor and none had received treatment.

### Immunohistochemistry

Immunohistochemistry was performed on the formalin-fixed and paraffin-embedded tissue sections using a standard immunohistochemical technique. Four-microgram-thick tissue sections were deparaffinized in xylene, rehydrated in a graded alcohol series, and treated with an antigen retrieval solution (10 mmol/L sodium citrate buffer, pH 6.0). The sections were incubated with rabbit monoclonal anti-AGR2 (Abcam, Cambridge, UK; dilution 1∶50), mouse monoclonal anti-CTSB (Abcam; dilution 1∶50) and mouse monoclonal anti-CTSD (Santa Cruz, dilution 1∶50) antibody overnight at 4°C. Subsequently, the sections were incubated with a biotinylated secondary antibody (Zhongshan, China), followed by incubation with an avidin–biotin complex (Zhongshan, China) according to the manufacturer’s instructions. Finally, tissue sections were incubated with 3′,3′-diaminobenzidine (DAB) (Sigma-Aldrich) and hydrogen peroxide for 2 min, and counterstained with hematoxylin for 30 s. In negative controls, primary antibodies were omitted.

### Evaluation of Staining

Sections were blindly evaluated by two investigators in an effort to provide a consensus on staining patterns under light microscopy (Olympus). AGR2, CTSB, and CTSD staining were assessed according to the methods described by Cheng et al. [56] with minor modifications. Each case was rated according to a score derived from a scale based on intensity of staining added to a scale based on area of staining. At least 10 high-power fields were chosen randomly, and >1000 cells were counted for each section. The depth of staining was graded on the following scale: 0, no cell coloration; 1+, light yellow; 2+, brown; 3+, tan. The area of staining was evaluated as follows: 0, no staining of cells in any microscopic fields; 1+, <30% of tissue stained positive; 2+, between 30% and 60% stained positive; 3+, >60% stained positive. The sum (extension+intensity) was used as the total score, where 0–1 indicates a negative score (−), ≥2 a positive score (+). Statistical analysis was performed using SPSS (version 18.0). A difference of *P*<0.05 was considered statistically significant.

### Immunofluorescence Analysis

The 6–10B cells treated with DNP were fixed with 2.0% formaldehyde in PBS for 30 min, washed with PBS three times, and then treated with PBS containing 0.2% Triton X-100 for 10 min. After being washed with PBS three times, the cells were incubated with 0.5% bovine serum albumin in PBS. The cells were incubated with AGR2 mouse antibody or cathepsin rabbit antibody, and respectively incubated with the anti-mouse antibody conjugated with FITC or anti-rabbit-IgG antibody conjugated with Texas Red after being washed. Cells were again washed using PBS, mounted onto coverslips, and examined under a Zeiss Axiophot microscope (Carl Zeiss, Oberkochen, Germany). Cells incubated with a non-specific IgG served as the blank control. Cells stained with DAPI served as the cell control.

### siRNA-AGR2 and Vectors

The siRNA-AGR2 was synthesized by Guangzhou RiboBio Limited Company (Guangzhou Guangdong China), and the sequence was as follows: 5′-CUGAUUAGGUUAUGGUUUATT-3′. When 30% confluence was reached, 6–10B cells were transfected with siRNA-AGR2 according to the manufacturer’s instructions. In parallel, cells transfected with nonspecific siRNA were used as a control. Control nonspecific siRNA was also purchased from Guangzhou RiboBio Limited Company of China. The pU6pro vector was used to construct pU6pro-si-mock (si-mock) and pU6prosi-AGR2 (si-AGR2) following the manufacture’s protocol. An AGR2 DNA fragment was generated by polymerase chain reaction (PCR) and cloned into the *Bam*HI/*Xho*I site of the pcDNA3.1 vector to generate pcDNA3.1-AGR2 plasmids.

### Gene Transfection and Stable Transfection of Cell

5–8F cells were transfected with U6pro-si-mock (si-mock) and pU6prosi-AGR2 using Lipofectamine 2000 reagent (Invitrogen), following the manufacturer’s suggested protocol. The stably transfected cell lines, 5–8F-si-mock, and 5–8F-si-AGR2 were obtained by selection for G418 resistance, and further confirmed by assessing AGR2 expression. 5–8F-si-AGR2 was further transfected with either pcDNA3.1 or pcDNA3.1-AGR2.

### Cell Invasion and Motility Assay

Cell invasion and motility assays were assayed according to the methods described previously with minor modifications [26]. For the invasion assay, untreated 6–10B cells, and 6–10B cells transfected with siRNA-AGR2 or nonspecific siRNA were treated with 8 μM DNP for 24 h. After DNP treatment, cells were removed by trypsinization, and their invasiveness was tested using the Boyden chamber invasion assay *in vitro*. Matrigel (25 mg/50 mL, Collaborative Biomedical Products, Bedford, MA) was applied to 8-mm pore size polycarbonate membrane filters. The treated cells were seeded into the upper part of the Boyden chamber (Neuro Probe, Cabin John, MD) at a density of 1.5×10^4^ cells/well in 50 μL of serum-free medium, and then incubated for 12 h at 37°C. The bottom chamber also contained standard medium with 20% FBS. The cells that invaded the membrane were fixed with methanol and stained with hematoxylin and eosin. Invaded cell numbers were counted under a light microscope. The motility assay was carried out as described for the invasion assay, but without Matrigel.

### Co-immunoprecipitation of AGR2 and CTSB or CTSD

For immunoprecipitation, 6.0×10^6^ 6–10B cells treated with DNP were lysed in 0.5 mL cell lysis buffer (20 mM Tris-HCl [pH 7.4], 150 mM NaCl, 1 mM EDTA, 1 mM EGTA, 1% Triton X-100, 2.5 mM sodium pyrophosphate, 5 mM beta-glycerophosphate, 1 mM Na_3_VO_4_, 1 μg/mL leupeptin, 1 mM phenylmethylsulfonyl fluoride, and 1 μM aprotinin) for 10 min at 4°C. After brief sonication, the cells were centrifuged at 12,000×*g* for 15 min at 4°C. One hundred micrograms supernatant was incubated overnight with 2 μg anti-AGR2 body (Abcam, rabbit monoclonal) and protein-G beads. The immunoprecipitates were collected and washed three times with RIPA buffer (50 mM Tris, pH7.5, 150 mM NaCl, 1% NP-40, 0.25% sodium deoxycholate, 1 Mm Na_3_VO_4_, 1 mM NaF, and protease inhibitor), and finally subjected to western blot analysis using anti-CTSB (Abcam, mouse monoclonal), or anti-CTSD (Santa Cruz, mouse monoclonal) antibody.

### Western Blotting

Western blotting analysis was performed as previously described [27]. Cell lysates and conditioned media were prepared, and protein concentrations were measured using Bio-Rad reagent (Bio-Rad). Cell lysates were denatured with an equal amount of 2×sample loading buffer by heating at 100°C for 10 min, and were then separated on a 10% polyacrylamide gel and transferred onto a nitrocellulose membrane (Bio-rad). The membranes were subsequently incubated with 5% non-fat milk in Tris-buffered saline containing 0.05% Tween-20 (TBST) for 1 h to block non-specific binding, and then incubated overnight with antibody against AGR2, CTSB, or CTSD (Cell Signaling Technologies). After being washed three times for 5 min each, the membranes were incubated with the secondary antibody for 1 h at room temperature. Finally, target proteins were detected by ECL (Pierce, Rockford, USA). Western blotting with anti-GAPDH antibody (Millipore) was used as an internal control to verify basal level expression and equal protein loading. The abundance ratio to GAPDH was measured.

## Results

### Expression of AGR2, CTSB, and CTSD in NNET, NPC, and Metastatic NPC

**Figure 1:**
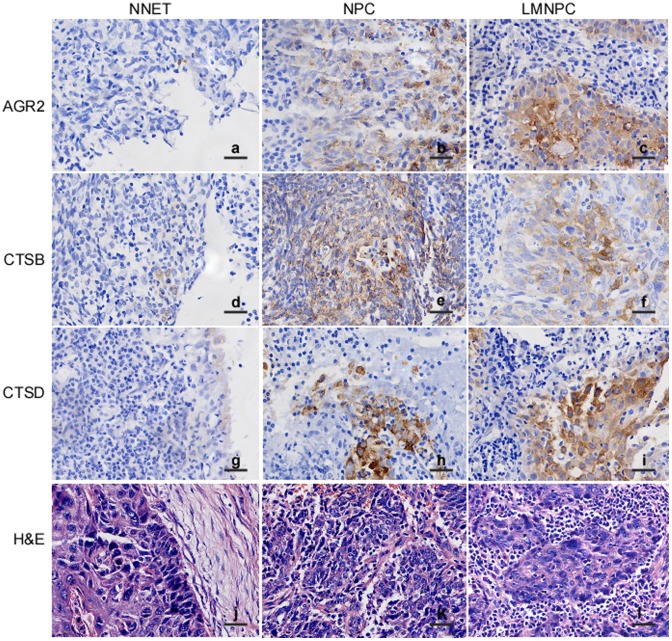
Immunohistochemical staining of AGR2, CTSB, and CTSD expressions in normal nasopharyngeal epithelial tissues (NNET), nasopharyngeal carcinoma (NPC), and cervical lymph node metastatic NPC (LMNPC) tissues. The formalin-fixed and paraffin-embedded NNET, NPC, and LMNPC tissue sections were stained using a standard immunohistochemical technique. a, AGR2 expression in NNET; b, AGR2 expression in NPC; c AGR2 expression in LMNPC; d, CTSB expression in NNET; e, CTSB expression in NPC; f, CTSB expression in LMNPC; g, CTSD expression in NNET; h, CTSD expression in NPC; i, CTSD expression in LMNPC; j, NNET hematoxylin and eosin; k, NPC hematoxylin and eosin; l, LMNPC hematoxylin and eosin. Arrows, positive cells. Original magnification, ×400. Scale bar, 5 μm.

AGR2, CTSB, and CTSD expression was detected using immunohistochemistry in formalin-fixed and paraffin-embedded archival clinical tissues, including 72 cases of NNET, 60 cases of NPC, and 78 cases of cervical LMNPC. The immunohistochemistry results revealed positive AGR2 (Fig. 1A–b, c), CTSB (Fig. 1a–e, f), and CTSD (Fig. 1a–h, i) signals showing brown-yellow granules in the cytoplasm, in the cytoplasm and membrane, and in the cytoplasm and membrane, respectively. Furthermore, as shown in Table 1, the percentage of positive staining in NNET tissues was 20.8%, 25.0%, and 30.5%; NPC tissues: 35.0%, 53.3%, and 36.7%; and LMNPC tissues: 53.8%, 48.7% and 58.9% for AGR2, CTSB, and CTSD respectively. AGR2, CTSB, and CTSD were significantly upregulated in LMNPC compared to NNET (*P*<0.01), and AGR2 and CTSD were also upregulated in LMNPC when compared to primary NPC (*P*<0.05), whereas no change of CTSB between LMNPC and primary NPC (*P*>0.05) was observed. All the results indicate that dysregulation of AGR2, CTSB, and CTSD might be related to development and metastasis of NPC.

**Table 1:** Differential AGR2, CTSB, and CTSD expression among NNET, NPC, and LMNPC tissues.

		AGR2				CTSB				CTSD			
	n	−	+	%	*P*	−	+	%	*P*	−	+	%	*P*
NNET	72	57	15	20.8	0.080*	54	18	25.0	0.001*	50	22	30.5	0.466*
NPC	60	39	21	35.0	0.038**	28	32	53.3	0.611**	38	22	36.7	0.011**
LMNPC	78	36	42	53.8	0.000***	40	38	48.7	0.004***	32	46	58.9	0.001***

Note: *, NNET versus NPC, **, NPC versus LMNPC, ***, LMNPC versus NNET.

### Expression of AGR2 is Associated with CTSB and CTSD in NPC Tissues

**AGR2, CTSB, and CTSD** are highly expressed in metastatic NPC, which implies that AGR2 is associated with CTSB or CTSD. The correlation of AGR2 expression with CTSB or CTSD was analyzed in NPC tissues. As shown in Table 2, AGR2 protein expression was positively correlated with CTSB (*r* = 0.145, *P*<0.05) and CTSD (*r* = 0.163, *P*<0.05). These data show that AGR2 and CTSB or CTSD are co-expressed in NPC tissues.

**Table 2:** Correlation of AGR2 with CTSB and CTSD expression in NPC and LMNPC tissues.

AGR2	CTSB				CTSD			
	−	+	r	P	−	+	r	P
**−**	60	40	0.145	0.035	66	39	0.163	0.018
+	50	60			49	56		

Note: *r* represents correlation coefficient.

### DNP Induces the Expression of AGR2, CTSB and CTSD

**Figure 2:**
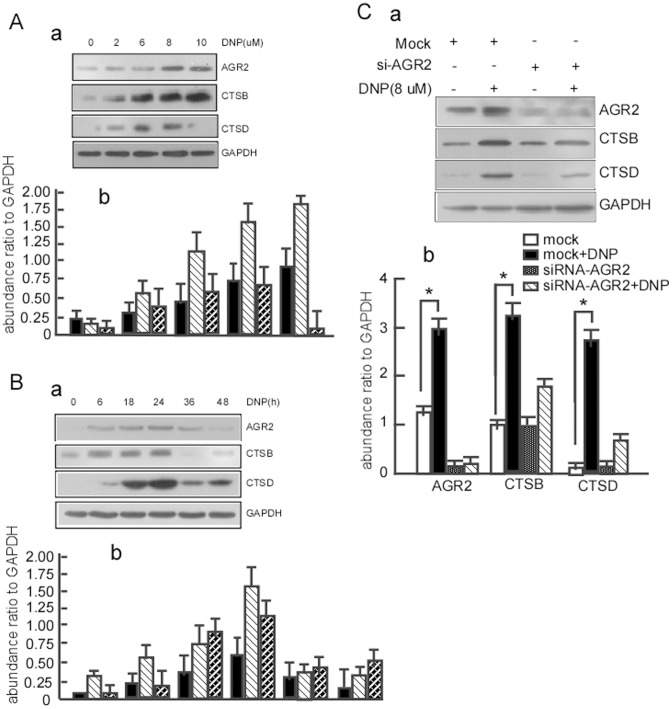
DNP induces expression of AGR2 and cathepsins B and D. A, 6–10B cells were treated with 8 μM DNP for the indicated time for a time-course. B, 6–10B cells were treated with the indicated concentration of DNP for a dose-course. AGR2, CTSB, and CTSD expression in the DNP-treated cells was detected using western blotting. GAPDH served as the loading control. C, 6–10B cells were treated with 8 μM DNP for 24 h. AGR2 was immunoprecipitated with anti-AGR2 antibody. CTSB and CTSD in the immunoprecipitations were then detected with western blotting. *indicates *P*<0.05. One representative experiment is presented.

AGR2, CTSB, and CTSD are highly expressed in metastatic NPC. A specific carcinogen for NPC, DNP is involved in NPC metastasis. The next step is to determine whether DNP contributes to high AGR2, CTSB, and CTSD expression. Our previous work showed significant changes in AGR2 levels at 24 hours after DNP treatment [27]. To confirm whether DNP can induce AGR2, CTSB, and CTSD expression, 6–10B cells were treated with 0–10 μM DNP for 24 h for the dose-course assay or with 8 μM DNP for 0–48 h for the time-course assay. AGR2, CTSB, and CTSD expression were then detected using western blotting. DNP induced AGR2 (Fig. 2A, lane 2, 3, 4, 5 *vs* 1 in 1^st^ panel a, and lane 4, 7, 10, 13 *vs* 1 in panel b), CTSB (Fig. 2A, lane 2, 3, 4, 5 *vs.* 1 in 2^nd^ panel a, and lane 5, 8, 11, 14 *vs.* 2 in panel b) and CTSD (Fig. 2A, lane 2, 3, 4 *vs.* 1 in 3^rd^ panel a, and lane 6, 9, 12 *vs.* 3 in panel b) expression in a time-dependent manner, and induced AGR2 (Fig. 2B, lane 2, 3, 4 *vs.* 1 in 1^st^ panel a, and lane 4, 7, 10 *vs.* 1 in panel b), CTSB (Fig. 2B, lane 2, 3, 4 *vs.* 1 in 2^nd^ panel a, and lane 5, 8, 11 *vs.* 2 in panel b) and CTSD (Fig. 2B, lane 2, 3, 4 *vs.* 1 in 3^rd^ panel a, and lane 6, 9, 12 *vs.* 3 in panel b) in a dose-dependent manner. These findings indicate that high AGR2, CTSB, and CTSD expression levels are induced by DNP.

The above AGR2 expression was associated with CTSB or CTSD in metastatic NPC tissues. Dumartin et al. reported that AGR2 regulates CTSB and CTSD to promote cell dissemination in cancer metastasis [35]. To further clarify whether DNP-mediated AGR2 regulates CTSB or CTSD expression, the untreated 6–10B cells and the 6–10B cells transfected with siRNA-AGR2 or with nonspecific siRNA, were treated with 8 μM DNP for 24 h, and then AGR2, CTSB, and CTSD expression was measured. First, AGR2 knockdown efficiency was examined using western blotting. AGR2 expression was significantly attenuated in AGR2 knockdown cells compared to the cells transfected with nonspecific siRNA (Fig. 2C-a, lane 3 *vs.* 1 in 1^st^ panel; Fig. 2C-b, lane 3 *vs.* 1 panel). In AGR2 knockdown cells, CTSB (Fig. 2C-a, lane 4 *vs.* 3 in 2^nd^ panel; Fig. 2C-b lane 8 *vs.* 7 panel) and CTSD (Fig. 2C-a, lane 4 *vs.* 3 in 3^rd^ panel; Fig. 2C-b lane 12 *vs.* 11 panel) levels did not change after DNP treatment. However, when AGR2 normally expressed, AGR2 (Fig. 2C-a, lane 2 *vs.* 1 in 1^st^ panel; Fig. 2C-b lane 2 *vs.* 1 panel), CTSB (Fig. 2C-a, lane 2 *vs* 1 in 2^nd^ panel; Fig. 2C-b lane 6 *vs.* 5 panel), and CTSD (Fig. 2C-a, lane 4 *vs.* 3 in 3^rd^ panel; Fig. 2C-b lane 10 *vs.* 9 panel) expression all increased after DNP treatment. The results show that DNP induces CTSB and CTSD expression through AGR2.

### DNP Increases the Interaction of AGR2 with CTSB and CTSD

**Figure 3:**
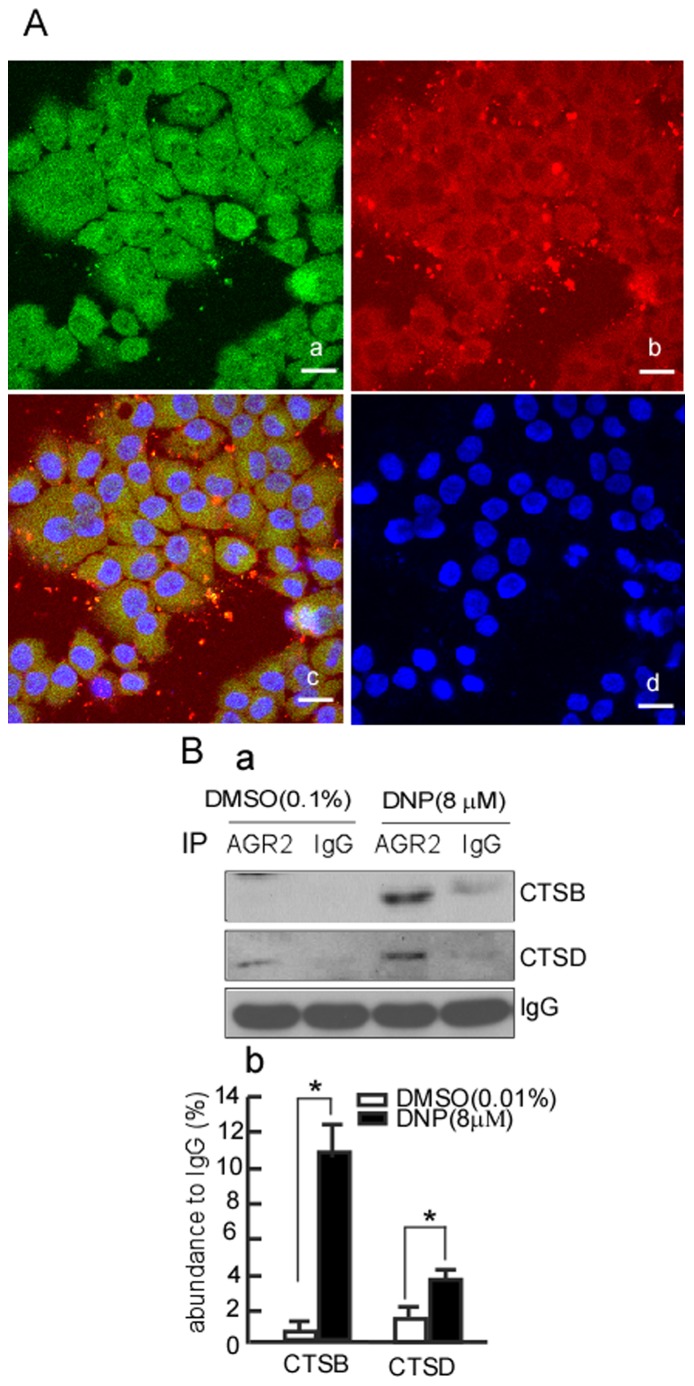
AGR2 interacts with CTSB/CTSD. A, interaction of endogenous AGR2 and CTS in 6–10B cells. Endogenous AGR2 and CTS expression in 6–10B cells was detected using an immunofluorescence assay. a, AGR2 expression in 6–10B cells; b, CTS expression in 6–10B cells; c, merge of a and b; d, DAPI staining. B, 6–10B cells were respectively transfected with siRNA-AGR2 or si-mock, and then treated with DNP. AGR2 was immunoprecipitated using AGR2 antibody, and CTSB and CTSD were detected in the immunoprecipitates using western blotting. IgG served as the loading control. *indicates *P*<0.05. One representative experiment is presented.

To determine the possibility of AGR2 binding to CTS, we detected the colocalization of AGR2 and CTS using the immunofluorescence assay. The results showed that AGR2 and CTS colocated in the cytoplasm (Fig. 3A–c). To further elucidate whether AGR2 binds to CTSB or CTSD using the immunoprecipitation assay, after DNP treatment, AGR2 was immunoprecipitated by AGR2 antibody, and then CTSB and CTSD were detected in the immunoprecipitates. The results showed that CTSB dramatically increased in the immunoprecipitates of the DNP-treated cells compared to the DNP-untreated group (Fig. 3B-a, lane 3 *vs.* 1 in 1^st^ panel; Fig. 3B-b lane 1 *vs* 2, *p*<0.05). CTSD also increased in the DNP-treated group (Fig. 3B-a, lane 3 *vs.* 1 in 2^nd^ panel; Fig. 3B-b lane 3 *vs.* 4, *p*<0.05). These results indicate that DNP induced binding of AGR2 with CTSD and CTSB.

### DNP-mediated Invasion and Motility Through Increased AGR2 Expression

**Figure 4:**
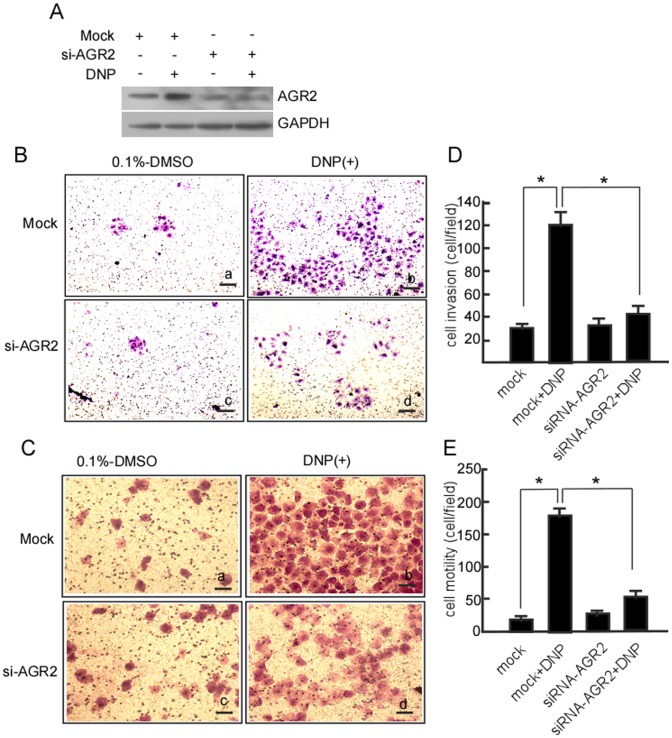
DNP-mediated invasion and motility through AGR2 in 6–10B cells. 6–10B cells were transiently transfected with siRNA-AGR2 or si-mock, and then treated with DNP. A, AGR2 expression was detected using western blotting. A Matrigel-coated Boyden chamber was used to measure 6–10B cell invasion, and an uncoated Boyden chamber was used to determine cell motility. The cells that invaded the membrane were fixed with methanol and stained with hematoxylin and eosin. Images were taken under a light microscope and random fields of view were counted to determine the number of invading cells. B, cell invasion of the treated cells. a, 6–10B cells transfected with si-mock and treated with 0.1% DMSO; b, 6–10B cells transfected with si-mock and treated with 8 μM DNP; c, the transfect with siRNA-AGR2 plus DMSO treatment; d, the transfect with siRNA-AGR2 and treated with DNP. C, cell motility of the treated cells. a, 6–10B cells transfected with si-mock and treated with DMSO; b, 6–10B cells transfected with si-mock and treated with DNP; c, the transfect with siRNA-AGR2 and treated with DMSO; d, the transfect with siRNA-AGR2 and treated with DNP. D, invasive cells were counted. E, motile cells were counted. The number of traversed cells were counted in three individual experiments and presented as the mean ± SD. *indicates *P*<0.05.

Our previous work has shown that DNP can induce NPC cell motility and invasion [27]. To confirm whether DNP-mediated metastasis occurs through AGR2, 6–10B cells were transfected with siRNA-AGR2 to block AGR2 expression, and then cell motility and invasion were measured. AGR2 was effectively silenced in 6–10B-si-AGR2 cells (Fig. 4A, lane 3 in upper panel). DNP-induced invasion (Fig. 4B–d; Fig. 4D lane 4 *vs.* 2, *P*<0.05) and motility (Fig. 4C–d; Fig. 4E, lane 4 *vs.* 2, *P*<0.05) were dramatically decreased when AGR2 expression was blocked. However, DNP could effectively induce cell invasion (Fig. 4B-b; Fig. 4D, lane 2 *vs.* 1, *P*<0.05) and motility (Fig. 4C-b; Fig. 4E lane 2 *vs.* 1, *P*<0.05) when AGR2 was not blocked. Taken together, our data indicate that DNP induces cell metastasis *via* induction of AGR2.

### si-AGR2 Decreased DNP-mediated Invasion and Motility in 5–8F Cells

**Figure 5:**
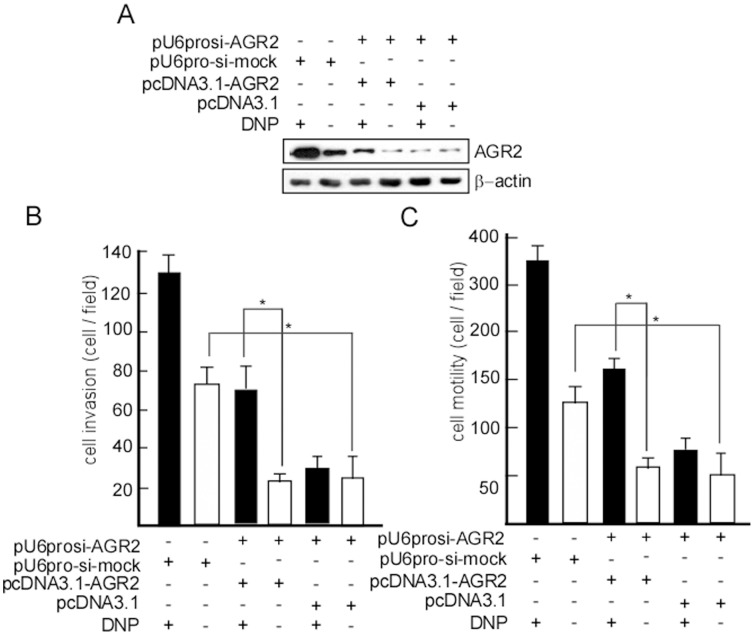
si-AGR2 decreased DNP-mediated invasion and motility in 5–8F cells. 5–8F cells were transfected with pUprosi-AGR2 (si-AGR2) or pUprosi-mock (si-mock), and 5–8F-si-AGR2 and 5–8F-si-mock were obtained by selection for G418 resistance, and treated with DNP. A, AGR2 expression was detected using western blotting. A Boyden chamber was used to measure the invasion and motility of 5–8F-si-AGR2 and 5–8F-si-mock with or without DNP treatment. The cells that invaded the membrane were fixed with methanol and stained with hematoxylin and eosin. Random fields of view were counted to determine the number of invading cells. B, invasion of the treated cells. C, motility of the treated cells. The number of traversed cells was counted in three individual experiments and presented as the mean ± SD. *indicates P<0.05.

To further confirm that AGR2 is involved in DNP-mediated metastasis, 5–8F, an NPC cell line with high metastatic ability, was transfected with pUprosi-AGR2 (si-AGR2), and 5–8F-si-AGR2 was obtained by selection for G418 resistance. AGR2 expression was blocked in 5–8F-si-AGR2 cells (Fig. 5A, lane 4, 5, 6 in up panel), and motility and invasion of 5–8F-si-AGR2 dramatically decreased (Fig. 5B, lane 6 *vs.* 2, *P*<0.05; Fig. 5C, lane 6 *vs.* 2, *P*<0.05). DNP could not effectively induce 5–8F-si-AGR2 motility and invasion (Fig. 5B, lane 4 *vs.* 5; Fig. 5C, lane 4 *vs.* 5). However, when we introduced AGR2, motility and invasion dramatically increased after DNP treatment (Fig. 5B lane 3 *vs.* 4, *p*<0.05; Fig. 5C lane 3 *vs.* 4, *p*<0.05).

## Discussion

In the clinic, NPC has a highly invasive and metastatic character, with approximately 87.9% of patients showing metastasis at initial diagnosis [67]. Well-established risk factors for NPC are genetic susceptibility, infection with the EBV, and regular consumption of salted fish beginning in childhood [2]. As an NPC carcinogen, EBV latent membrane protein-1 and -2 (LMP1 and LMP2) have been confirmed to promote progression and metastasis of NPC, but the positive expression rate of EBV in NPC tissues is only 57% [58], [59]. Presently, we focused on the carcinogen, DNP.

The process of salt preservation is inefficient, allowing fish and other foods to become partially putrefied [16], [60]. Bacteria induce the conversion of nitrates into nitrites, which form important carcinogenic *N*-nitroso compounds [60]. These foods accumulate significant levels of nitrosamines [16], [17], [19], [61]. When salted foods are consumed, nitrosamines are transformed into DNP in the liver [13], [20], [62], and DNP is a predominant volatile nitrosamine. DNP has been shown to participate in NPC development. In experiments concerning DNP-induced rat NPC, DNP showed organ specificity for the nasopharyngeal epithelium and a high incidence of NPC metastasis [25], [62]. In clinical assays, NPC patients with metastasis have high levels of DNP [26].

In our previous works, to reveal the pathogenesis of DNP, a quantitative proteomic study using stable isotope labeling with amino acids in cell culture (SILAC), coupled with mass spectrometry, was performed on DNP-treated 6–10B cells. Many protein showed significant changes in levels at 24 h after DNP-treatment, including AGR2, CTSB, and CTSD [27]. Recently, much interest has focused on AGR2 for its potential roles in the invasion and metastasis of malignant tumors [63]. AGR2 is frequently overexpressed in many human cancers, including breast carcinoma [28], lung adenocarcinoma [29], [30], ovarian carcinoma [31], and prostate cancers [32]. In the present study we also found that positive expression of AGR2 in NPC tissues was significantly higher than in the normal nasopharyngeal tissues. Further, we analyzed the correlation of AGR2 expression with CTSB or CTSD in NPC tissues. It has been reported that overexpression of AGR2 is associated with poor differentiation, deep invasion, and lymph node metastasis in several types of cancer [32], [34], [64], [65]. Surprisingly, our data demonstrated overexpression of AGR2 was associated with lymph node metastasis in NPC tissues as well. Taken together, the above results suggest that AGR2 acts as a pivotal factor contributing to the progression of NPC, and may be involved in the invasion and metastasis of NPC.

Our previous work has shown that DNP can induce NPC cell motility and invasion [27]. To confirm whether DNP-mediated metastasis occurs through AGR2, AGR2 was silenced in 6–10B cells. Consequently, DNP-mediated motility and invasion of 6–10B cells was decreased. These results were confirmed in 5–8F cells. DNP did not have an effect on 5–8F cell motility and invasion when AGR2 was blocked. On the other hand, AGR2 gene overexpression significantly increased CTSB and CTSD expression [35]. CTSB and CTSD contribute to tumor cell invasion and angiogenesis and are commonly associated with metastasis [38], [46], [48], [52]. Cathepsin is known to remodel the surrounding ECM by proteolysis, allowing tumor cell invasion and metastasis [66]. In the current study, we demonstrated that AGR2 coexpresses with CTSB/CTSD in NPC cells and tissues, and that downregulation of AGR2 by siRNA results in the reduction of the CTSB/CTSD expression in 6–10B cells with DNP treatment. This suggests that activation of CTSB/CTSD is regulated by the AGR2 gene in DNP-mediated metastasis. On the other hand, immunoprecipitation assays have shown that DNP increases the interaction of AGR2 and CTSB. We speculate that a possible mechanism of AGR2-induced NPC metastasis may be the remodeling of the extracellular matrix (ECM) through CTSB and CTSD.

## Conclusion

AGR2 is an important factor in the invasion and metastasis of NPC. It may exert control, at least in part, through the regulation of CTSB and CTSD. DNP induces AGR2 expression, and DNP-mediated AGR2 stimulates the production of CTS from endothelial cells, thereby stimulating the remodeling of ECM and accelerating tumor invasion and metastasis. Additionally, DNP-mediated AGR2 and CTSB/CTSD expression involving NPC metastasis provides new avenues for the high incidence of NPC metastasis.
